# Supplementary material for: Content-rich biological network constructed by mining PubMed abstracts
Source: BMC Bioinformatics. 2004 Oct 8;5:147. doi: 10.1186/1471-2105-5-147 (PMC528731; doi:10.1186/1471-2105-5-147)
Supplement: Additional File 5 — The original Chilibot query results of the term "long-term potentiation (LTP)" and 22 other terms, limiting the latest references analyzed to the years 1990, 1995, 2000, and 2004. [file 1471-2105-5-147-S5.bz2 › chilibotAdditionalFile5/ltp2000/html/PI-3K_ZIF268.html]

 


 **PI-3K** and **ZIF268** 
  
Found 1 abstracts in PubMed,  **1 abstracts were retrieved and analyzed**.  


---

 Search Google  |
 PDF files only 
|  EDU domain only 

---

**Interactive relationship** (e.g. stimulation, inhibition, etc)

- Together, these results suggest that MAPK, PI 3 kinase  [ **PI-3K** ]  and JNK each play a role in the induction of  **zif268**  gene expression by NGF in PC12D cells.  Ref: 10050043 J Biochem (Tokyo), 1999
- Much of the residual induction of  **zif268**  mRNA is blocked by low concentrations of wortmannin, an inhibitor of phosphatidylinositol PI 3 kinase  [ **PI-3K** ] .  Ref: 10050043 J Biochem (Tokyo), 1999

- :-)

  **Parallel relationship** (e.g. studied together, co-existance, homology, etc.)

  - Stimulation of PC12D cells with NGF weakly activates JNK, but this activation is enhanced rather than inhibited by pretreatment with wortmannin, suggesting that JNK does not function downstream of PI 3 kinase  [ **PI-3K** ]  in the induction of  **zif268**  mRNA.  Ref: 10050043 J Biochem (Tokyo), 1999
  - Since PI 3 kinase  [ **PI-3K** ]  was previously shown to function upstream in epidermal growth factor EGF mediated activation of c Jun N terminal kinase JNK, and JNK is known to phosphorylate and activate transcription factors that regulate the expression of  **zif268** , we investigated the role of JNK in the induction of  **zif268**  mRNA by NGF.  Ref: 10050043 J Biochem (Tokyo), 1999
